# Augmenting intensive care unit nursing practice with generative AI: A formative study of diagnostic synergies using simulation‐based clinical cases

**DOI:** 10.1111/jocn.17384

**Published:** 2024-08-05

**Authors:** Chedva Levin, Moriya Suliman, Etti Naimi, Mor Saban

**Affiliations:** ^1^ Nursing Department, Faculty of School of Life and Health Sciences The Jerusalem College of Technology‐lev Academic Center Jerusalem Israel; ^2^ Department of Vascular Surgery The Chaim Sheba Medical Center Ramat Gan Tel Aviv Israel; ^3^ Intensive Care Unit The Chaim Sheba Medical Center Ramat Gan Tel Aviv Israel; ^4^ Department of Nursing, School of Health Professions, Faculty of Medical and Health Sciences Tel Aviv University Tel Aviv Israel

**Keywords:** clinical scenarios, decision‐making, diagnostic accuracy, generative artificial intelligence, intensive care units

## Abstract

**Background:**

As generative artificial intelligence (GenAI) tools continue advancing, rigorous evaluations are needed to understand their capabilities relative to experienced clinicians and nurses. The aim of this study was to objectively compare the diagnostic accuracy and response formats of ICU nurses versus various GenAI models, with a qualitative interpretation of the quantitative results.

**Methods:**

This formative study utilized four written clinical scenarios representative of real ICU patient cases to simulate diagnostic challenges. The scenarios were developed by expert nurses and underwent validation against current literature. Seventy‐four ICU nurses participated in a simulation‐based assessment involving four written clinical scenarios. Simultaneously, we asked ChatGPT‐4 and Claude‐2.0 to provide initial assessments and treatment recommendations for the same scenarios. The responses from ChatGPT‐4 and Claude‐2.0 were then scored by certified ICU nurses for accuracy, completeness and response.

**Results:**

Nurses consistently achieved higher diagnostic accuracy than AI across open‐ended scenarios, though certain models matched or exceeded human performance on standardized cases. Reaction times also diverged substantially. Qualitative response format differences emerged such as concision versus verbosity. Variations in GenAI models system performance across cases highlighted generalizability challenges.

**Conclusions:**

While GenAI demonstrated valuable skills, experienced nurses outperformed in open‐ended domains requiring holistic judgement. Continued development to strengthen generalized decision‐making abilities is warranted before autonomous clinical integration. Response format interfaces should consider leveraging distinct strengths. Rigorous mixed methods research involving diverse stakeholders can help iteratively inform safe, beneficial human‐GenAI partnerships centred on experience‐guided care augmentation.

**Relevance to Clinical Practice:**

This mixed‐methods simulation study provides formative insights into optimizing collaborative models of GenAI and nursing knowledge to support patient assessment and decision‐making in intensive care. The findings can help guide development of explainable GenAI decision support tailored for critical care environments.

**Patient or Public Contribution:**

Patients or public were not involved in the design and implementation of the study or the analysis and interpretation of the data.

**What does this paper contribute to the wider global community?**

- This study contributes new knowledge on the diagnostic performance of ICU nurses compared to leading GenAI systems through objective assessments of standardized and open‐ended clinical cases, providing insights into current GenAI capabilities and limitations for replicating expert clinician cognition.
- The findings underline the importance of continued research towards judiciously integrating human experience with evolving GenAI capabilities to enable enhanced clinical decision‐making as technologies advance. The results generate knowledge that can help guide the prudent development and evaluation of GenAI‐enabled decision support tools tailored specifically for ICU environments and practitioners.

## 1 INTRODUCTION

**FIGURE 1:**
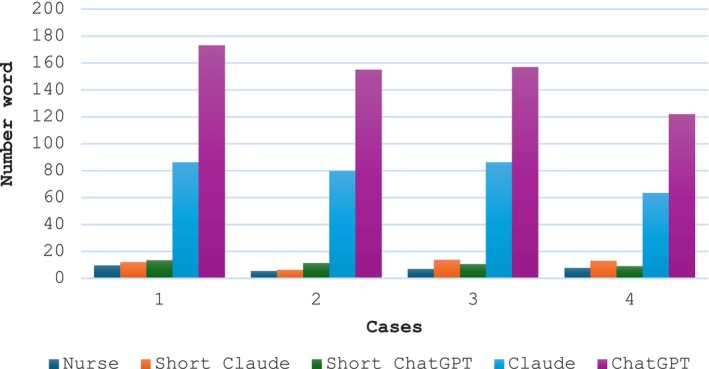
Significant differences in the number of words in the nurses' responses compared to the four major LLM language types for each of the four cases (Case 1: *F* = 281, *p* = .00, case 2. *F* = 500.6, *p* = .00; case 3: *F* = 398, *p* = .00, case 4: *F* = 352, *p* = .00). The nurses used the least number of words compared to the full ChatGPT, which used the highest number of words. For example, in the fourth case that dealt with bleeding, the average number of words in a nurse's response was 9.6 ± 4.72, versus 173 words for the full ChatGPT, 86.2 words for Claude, 64.5 words for the short ChatGPT and 50.4 words for the short Claude. [Colour figure can be viewed at wileyonlinelibrary.com]

**FIGURE 2:**
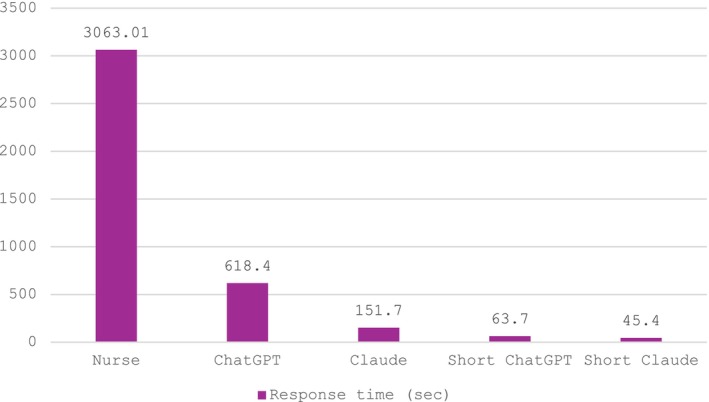
Significant differences in the reaction times to solve all four cases between nurses and the four types of large language models (LLMs) (*F* = 18.12, *p* = .00). The response time of nurses was notably more than 60 times higher compared to the short models, 20 times higher than the full Claude and 5 times higher than the full ChatGPT. [Colour figure can be viewed at wileyonlinelibrary.com]

Accurate and rapid clinical decision‐making (CDM) is vital in the intensive care unit (ICU) where patients often present with complex, life‐threatening conditions involving multi‐organ failure (Gopalan & Pershad, 2019). Clinicians in the ICU are responsible for providing comprehensive care to patients with complex, life‐threatening conditions involving multiple organ systems (Figures 1 and 2). This requires continuous monitoring, assessment, prioritization, diagnosis, treatment decisions and care coordination. CDM therefore represents a core function and driving force behind daily ICU clinical workflows and activities (James et al., 2018).

The CDM process encompasses many interrelated components, including gathering a thorough history and performing a comprehensive physical exam, interpreting diagnostic test results, formulating a differential diagnosis, weighing treatment options while considering patients' values and preferences, re‐evaluating plans as new information emerges and effectively communicating with patients, families and the interprofessional care team (Saintsing et al., 2011; Nibbelink & Brewer, 2018). Due to its all‐encompassing, analytic nature, CDM draws upon higher‐order cognitive skills such as critical thinking, clinical reasoning and sound clinical judgement (Nursing PBATAJ of 1982, 2023).

While experience enhances CDM capabilities over time, challenges remain particularly for less experienced ICU clinicians (Archibald & Clark, 2023; Jin & Ji, 2021; Wong & Kowitlawakul, 2020). Novice clinicians in the ICU and new graduates may struggle with various aspects of the decision‐making process, due to lack of exposure to complex clinical cases and limited intuition developed from years of practice. This introduces risks if novices practice independently without adequate support. Existing research demonstrates deficiencies in CDM skills among novice clinicians compared to experts.

Concurrently, generative artificial intelligence (GenAI) models powered by large language models (LLMs) have advanced rapidly and demonstrate human‐level capacities for natural language generation (Gaube et al., 2021; Huh, 2023; Kao et al., 2023; Saban & Dubovi, 2024). Chat‐based GenAI tools such as ChatGPT utilize massive language models to understand clinical scenarios described in natural language and produce responses (Rosen & Saban, 2023a; 2023b). While initially developed for other domains, preliminary work exploring ChatGPT's performance on clinical tasks shows promise to potentially support elements of CDM through natural dialogue, if applied judiciously (Alkhaqani, 2023; Bajaj et al., 2023; Lyu et al., 2023; Miao & Ahn, 2023; Rosen & Saban, 2023a; 2023b). Claude is an GenAI agent created by Anthropic to assist users helpfully through a technique called Constitutional GenAI. With the ability to rapidly search medical information, Claude shows promise as a supplementary clinical decision support tool to help practitioners evaluate patients and treatment guidelines, pending rigorous validation of its reliability and safety (Borji & Mohammadian, 2023; Wu et al., 2023).

However, the ICU environment amplifies the importance of robust CDM due to critically ill patients with complex, life‐threatening conditions requiring rapid assessment and treatment decisions model. Novel educational techniques have attempted to address gaps in CDM training by supplementing real experiences. Simulation‐based learning simulates clinical scenarios in a controlled environment without risks to patients, overcoming scale limitations and unpredictability of clinical exposure. However, barriers still prohibit comprehensive CDM training. Current simulations remain narrow and lack rigorous evaluation against experts to identify opportunities for augmenting human performance. While authentic for learners, direct patient care also poses ethical issues and safety risks if used for novice training (Bahrani et al., 2013; Jee et al., 2023). Opportunities are constrained by limited resources as patient volumes, acuity and unpredictable schedules may not facilitate diverse encounters for mastery. Additional barriers include privacy regulations restricting use of identifiable patient data for education. Simply relying on real‐world experiences fails to standardize training or ensure comprehension of critical domains rarely seen (Gaba, 2007).

This study aimed to provide preliminary insights into how emerging GenAI technologies, such as large language models, could potentially interface with and support intensive care clinicians' CDM skills development and patient care optimization. Specifically, we conducted a comparative evaluation of GenAI and experienced clinician responses to simulated ICU case studies. The objectives were to (1) explore similarities and differences in human and AI clinical reasoning approaches; (2) understand the theoretical potential of integrating such technologies into practice, rather than eliciting direct clinician perspectives; and (3) identify opportunities and challenges for judicious partnerships between humans and GenAI focused on patient safety and care quality.

To rigorously assess current capabilities, our study focused on analysing the diagnostic accuracy and recommendations provided by two state‐of‐the‐art GenAI systems, ChatGPT‐4 and Claude‐2.0, in response to fundamental clinical questions derived from ICU patient scenarios. We then directly compared the GenAI performance to responses from certified critical care nurses with extensive ICU experience, analysing key decision‐making processes. By investigating feasible integration models from the outlook of frontline end‐users themselves, we ultimately aim to inform guidelines for developing and evaluating AI‐augmented clinical support tailored appropriately for ICU environments and practitioners.

## 2 METHODS

### 2.1 Design

The cross‐sectional study was conducted in October–December 2023, using a survey instrument that included four ICU clinical scenarios.

### 2.2 Participants

Participants consisted of three groups: (1) Academic nurses who actually work in ICUs (general intensive care, surgical/internal, cardiac intensive care, cardiovascular surgery intensive care, neurosurgical intensive care), about 40% of them have a master's degree and more than two‐thirds of them have unique basic training in intensive care; (2) ChatGPT‐4; and (3) Claude‐2.0. Collected sociodemographic data encompassed gender, age, general seniority, ICU experience, academic status and department type.

### 2.3 Sample selection method

In this formative study, a purposive sampling method was employed to select expert ICU nurses who were appropriate for evaluating performances. The selection process carefully considered specific qualifications and experience in ICU care. The aim was to ensure that the chosen nurses possessed the necessary expertise to provide valuable insights into the study objectives.

A total sample size of 74 ICU nurses from various regional hospitals was included to enhance the diversity of perspectives. The nurses selected had graduate degrees related to critical care, further establishing their appropriateness for evaluating performances within the context of the study. By employing purposive sampling, this study ensured that the selected ICU nurses were well suited to provide informed responses and contribute meaningfully to achieving the study's objectives. Their expertise and qualifications in critical care provided a solid foundation for evaluating the diagnostic synergies using simulation‐based clinical cases in the context of augmenting ICU nursing practice with GenAI (Campbell et al., 2020).

### 2.4 Procedure and data collection

The clinical reasoning questionnaire, featuring four clinical scenarios, was administered to nurses. Concurrently, ChatGPT‐4 and Claude‐2.0 were tasked with providing initial assessments and treatment recommendations for the same scenarios. (Appendix S1 presents the example questionnaire prompt used in the first case).

Each AI responded twice: once without word limits and again with a 10‐word constraint (referred to as the ‘Short Version’). Nurses completed the questionnaire via the web‐based platform Qualtrics XM. To assess ChatGPT‐4's clinical reasoning, we utilized the OpenAI Playground system, which generates unrestricted text responses. For evaluating Claude‐2.0's clinical reasoning, we utilized the Poe system (@poe), an AI bot developed by Anthropic for natural conversations. It facilitates safe, transparent interactions with Anthropic's Constitutional artificial intelligence technology.

The responses provided by nurses, ChatGPT‐4 and Claude‐2.0 were analysed for clinical reasoning, as outlined below.

Scenarios and assessment task development.

The development of the clinical scenarios was a meticulous process, designed to ensure that they accurately represented real‐life ICU situations. The scenarios were crafted by the two authors of the article who are certified ICU nurse practitioners. Their extensive experience and knowledge in ICU care were instrumental in creating scenarios that mirrored the complexity and ambiguity often encountered in real‐life ICU settings.

The scenarios were based on classic care reasoning and were developed in accordance with literature accepted in intensive care, particularly drawing from ‘Critical Care Nursing: A Holistic Approach’ (Merchant et al., 2020; Morton & Thurman, 2023). This ensured that the scenarios were grounded in established and accepted practices in ICU care.

Each case was deliberately designed to introduce ambiguity, presenting two possible diagnoses, and covered various clinical domains in ICU care: Case 1—CPR cardiopulmonary resuscitation; Case 2—Head Injury; Case 3—Diabetic Ketoacidosis; Case 4—Hemorrhagic shock.

Two nurses with advanced academic degrees in nursing (PhD and MA) validated all cases. The clinical scenarios included comprehensive details regarding comorbidity, clinical signs and symptoms mirroring real‐life ICU scenarios. For each scenario, participants were tasked with providing an initial evaluation. Subsequently, they were prompted to interpret and explain the tests the patients underwent. Following these responses, participants progressed to the second phase, where additional patient information was provided. Upon receiving this detailed patient information, participants were queried about the required care for the patients.

Clinical reasoning was evaluated for both the two LLMs and the ICU nurses based on three criteria: (i) the accuracy of evaluating resources, including laboratory or imaging tests; (ii) the accuracy of treatment decisions following the assessment of clinical test results along with additional data provided in the second stage of the study; and (iii) performance in clinical judgement, assessed by response time to all cases and the word count of responses to each case.

### 2.5 Assessment tools

The assessment tools used in this study were designed to evaluate the clinical reasoning of both the ICU nurses and the AI models. The tools focused on three criteria: (i) the accuracy of evaluating resources, including laboratory or imaging tests; (ii) the accuracy of treatment decisions following the assessment of clinical test results along with additional data provided in the second stage of the study; and (iii) performance in clinical judgement, assessed by response time to all cases and the word count of responses to each case.

The use of different experts for scenario development and actual assessment was a strategic decision made to ensure objectivity and validity in the study. The experts who developed the scenarios had a deep understanding of ICU care and were able to create complex and realistic scenarios. However, to avoid any potential bias, different experts were used to assess the responses. The assessors were also highly qualified ICU nurses, but they were not involved in the scenario development process. This ensured that they could objectively evaluate the responses based on the established criteria without any preconceived notions about what the responses should be. This separation of roles enhanced the reliability and validity of the study's findings.

### 2.6 Data analysis

Both authors jointly coded all responses. Clinical decision performance for each case scenario was evaluated using scores ranging from 0 to 100. Expert nurses independently scored each response based on diagnosis accuracy and management plan appropriateness, with final mean scores derived from the averaged scores of both nurses. Response time for each system (nurses vs. LLMs) was recorded from presentation of case details to final response generation. Mean response times were calculated for all cases.

Word counts in written responses for each case were tallied using an automated tool. Mean word counts and standard deviations were calculated for each system across all case responses. The inclusion of word count as an evaluation criterion in the context of generative GenAI tools is significant due to its implications for content depth and user engagement. A higher word count often signifies more detailed and comprehensive responses, demonstrating the GenAI's ability to provide extensive information on a topic. This can lead to more effective user engagement, particularly when complex concepts or detailed explanations are required. However, it's also crucial to maintain a balance as excessively long responses could potentially overwhelm users or make the conversation less interactive (Abbasian et al., 2024; Levin et al., 2024; Saban & Dubovi, 2024).

Descriptive statistical analyses utilized means and standard deviations for continuous variables and frequencies and percentages for categorical variables. ANOVA were employed for mean comparisons when appropriate, with significance set at *p* < .05.

We also implemented text analysis methodology that encompassed both qualitative and quantitative dimensions. Although the findings were quantitatively articulated, they underwent a qualitative interpretation process. This process incorporated content analysis, categorizing responses based on their succinctness or verbosity, and thematic analysis, identifying prevalent themes or patterns within these categories.

Statistical analyses were conducted using SPSS Version 28 software.

### 2.7 Ethical considerations

Prior to commencement, the study obtained approval from the university's ethics committee (#0006223‐2). All data collection procedures ensured anonymity. Nurses offered informed consent before participating, with the assurance of their right to withdraw from the study at any point and for any reason.

## 3 RESULTS

A total of 74 academic nurses employed in intensive care settings took part in the study, with 30 holding a master's degree (40.5%). The majority (68.3%) were primarily engaged in general intensive care, particularly internal or surgery. A significant proportion (82.4%) of these nurses had received specialized professional training for ICUs. The mean age of all participants was 38.8 ± 9.4 years, ranging from 25 to 64, with 73% being women. The average length of professional experience was 11.6 ± 9.9 years, varying from one to 40 years. In terms of ICU experience, participants had an average of 8.4 ± 9.2 years, with the range also spanning from one to 40 years. Table 1 provides an overview of the characteristics of study participants.

**TABLE 1:** Sociodemographic characteristics of the study sample (*N* = 74).

Variable		Mean (SD)
Age (25–64)		38.84 (9.49)
Seniority (1–40)		11.67 (9.91)
Seniority in ICU (1–40)		8.47 (9.24)

Variable		*N* (%)
Gender		
Male		20 (27)
Female		54 (73)
Academic status		
B. A		44 (59.5)
M.A		30 (40.5)
Training in intensive care	Yes	61 (82.4)
Type of intensive care unit		
General intensive care (surgical/internal)		51 (68.9)
Cardiac intensive care		12 (16.2)
Cardiovascular surgery intensive care		8 (10.8)
Neurosurgical intensive care		3 (4.1)

Table 2 reveals that nurses consistently achieved higher accuracy scores compared to LLMs across all scenarios except for resuscitation, where the full ChatGPT demonstrated the highest proficiency. Conversely, in the remaining three scenarios, the full ChatGPT exhibited the lowest scores, ranging from 13.8% to 40%.

**TABLE 2:** Clinical decision‐making performance scores (mean and standard deviation) for nurses and large language models across four intensive care units case scenarios.

Case	Nurse		Claude		Short claude		ChatGPT		Short ChatGPT		*F* score	*p* value
	Score for each question	Final score	Score for each question	Final score	Score for each question	Final score	Score for each question	Final score	Score for each question	Final score		
Case 1: Cardiopulmonary resuscitation	86.4 (34.4)	57.48 (16.3)	100	50	100	45	100	75	100	53.25	.50	.73
	86.4 (34.4)		50		80		100		33			
	86.4 (34.4)		50		0		100		50			
	86.4 (34.4)		0		0		0		30			
Case 2: Head injury	80.4 (22.9)	73.83 (14.4)	100	71.42	50	53.57	50	28.57	25	42.85	3.93	.00
	80.4 (22.9)		100		100		100		100			
	80.4 (22.9)		100		100		0		100			
	80.4 (22.9)		25		75		0		25			
	80.4 (22.9)		75		50		50		50			
	80.4 (22.9)		100		0		0		0			
	80.4 (22.9)		0		0		0		0			
Case 3: Diabetic ketoacidosis	70.9 (45.1)	77.64 (21.9)	80	68.8	0	55	0	13.83	80	16	4.15	.00
	70.9 (45.1)		100		80		0		0			
	70.9 (45.1)		100		50		0		0			
	70.9 (45.1)		100		100		50		0			
	70.9 (45.1)		33		100		33		16			
	70.9 (45.1)		0		0		0		0			
Case 4: Haemorrhagic shock	36.2 (18.9)		50	56.6	50	50.6	25	40	25	64.6		
	36.2 (18.9)		100		100		100		100			
	36.2 (18.9)		0		0		0		100			
	36.2 (18.9)		33		33		75		33			
	36.2 (18.9)		100		70		0		65			
*F* score/*p* value	28.4/.00		7.34/.00		2.15/.00		1.08/.00		1.24/.00			

Significant variations in accuracy levels were observed among the different models and nurses across the four scenarios. Nurses and the short ChatGPT excelled particularly in the fourth scenario involving bleeding, whereas the full Claude demonstrated higher accuracy in the second scenario concerning head injury. The short Claude showed superior accuracy in the third scenario on diabetic ketoacidosis. Notably, when solving the resuscitation scenario, no significant difference in accuracy was identified between nurses and LLM. However, significant differences were evident in the head injury, diabetic ketoacidosis and bleeding scenarios, wherein nurses achieved higher accuracy than LLMs.

No correlation was found between the clinical accuracy of the nurses' responses to all case scenarios and the socio‐demographic variables of gender, age, their professional academic status, general and professional seniority, and training in an intensive care course. Also, no relationship was identified between the type of ICU where the nurses work and the degree of clinical accuracy across the scenarios.

## 4 DISCUSSION

The findings from this study provide an important foundation for understanding how GenAI tools may interface with CDM in intensive care. While current LLMs demonstrated capabilities in certain standardized cases, experienced ICU clinicians consistently exhibited superior diagnostic accuracy and judgement across diverse open‐ended scenarios. These results align with prior research that also found GenAI tools outperformed on narrowly defined tasks but had more mixed performance on open‐ended clinical reasoning compared to experts (Saban & Dubovi, 2024). As clinical practice in the ICU involves complex, time‐sensitive situations with wide variability between patients, generalizability remains a significant challenge for GenAI, as others have similarly concluded. Unlike previous studies, in the current study, no relationship was found between professional seniority, specific training for work in the ICU and seniority in the ICU to the degree of accuracy in solving the scenarios. This lack of correlation could be attributed to the scenarios featuring common situations governed by structured and uniform protocols, thus allowing younger staff to perform on par with their more experienced counterparts (Archibald & Clark, 2023; Jin & Ji, 2021; Wong & Kowitlawakul, 2020).

However, GenAI tools also showed potential benefits like rapid responses that could help support workforce strains experienced by ICUs, echoing suggestions from the literature that GenAI may help address clinician shortages if used judiciously (Barash et al., 2023; Johnson et al., 2023; Rosen & Saban, 2023b; Tam et al., 2023). If advanced judiciously while acknowledging their limitations, GenAI assistants may play meaningful roles in bolstering overextended clinicians and aiding novice practitioners' skills development, supporting the aims of prior studies. By simulating expert reasoning approaches via vast training datasets, GenAI tutoring holds promise to expand high‐fidelity decision‐making training availability, consistent with the aims of simulation‐based trainings explored in other work.

Our research, which draws upon the principles of the Turing Test, serves as a unique exploration within the healthcare sector. The Turing Test, introduced by Alan Turing, is a method used to determine if a machine can exhibit intelligent behaviour that is equivalent or indistinguishable from human behaviour (Sejnowski, 2023).

Our results indicate that while LLMs can replicate some human behaviours, such as providing rapid responses, they are deficient in other crucial areas. For example, nurses consistently surpassed LLMs in accuracy across a variety of scenarios, with resuscitation being the sole exception. This suggests that LLMs' capacity to mimic human‐like clinical reasoning is still limited (Aharoni et al., 2024).

Another facet of the Turing Test is the generation of responses that are not only precise but also succinct and efficient. In this context, our study reveals that nurses employed fewer words than LLMs, demonstrating a more effective communication style. This underscores the existing limitations of LLMs in producing concise and pertinent responses (Kirova et al., 2023; Zhang & Kamel Boulos, 2023).

Overall, the results validate concerns raised in previous analyses underscoring that GenAI is not yet positioned to replace core elements of human clinical expertise. One of the most notable aspects of the GenAI tool is its use of a large number of words, which can obscure the essential steps required for effective clinical decisions. In a metaphorical sense, it can be likened to ‘You can't see the forest for the trees’ due to its excessive wordiness. However, GenAI could play an augmentative role if risks are mitigated, as recommended by related ethical and standards frameworks. Looking ahead, continued interdisciplinary collaboration aligning with calls for multistakeholder research partnerships in this area needed to iteratively refine GenAI evaluation methodologies, address generalizability challenges, ensure GenAI recommendations are transparent and remain under human supervision. With informed integration supported by rigorous research and stakeholder perspectives, GenAI may help advance the goals of judicious augmentation explored in prior investigations to assist ICU clinicians to manage complex patients more effectively while upholding patient‐centred care as the top priority.

Additionally, interrater‐reliability is an important consideration when evaluating the performance of both human clinicians and GenAI tools. Assessing the level of agreement among multiple raters in evaluating clinical scenarios can provide insights into the consistency and reliability of their judgements. Interrater‐reliability analysis will contribute to the overall assessment of the reliability and robustness of diagnostic and decision‐making processes in the ICU. It will help identify areas of consensus or variability among experts, shedding light on potential discrepancies or inconsistencies and highlighting areas for further training or improvement (Huang et al., 2023; Zaretsky et al., 2024).

While this study focused on comparing the performance of ICU nurses and GenAI tools, future research could explore interrater‐reliability by involving multiple expert clinicians in the evaluation process.

### 4.1 Limitation

There are some limitations to note in this preliminary study. First, the simulated case scenarios represent a narrow subset of clinical complexity compared to real‐world patient presentations. Performance on simulations may therefore overestimate capabilities for diverse clinical encounters. Second, the relatively small sample size of nurses from a single region may limit generalizability. Including more nurses from varied backgrounds could provide more robust comparisons. Third, quantitative accuracy metrics alone cannot fully capture the nuanced qualitative aspects of clinical reasoning, such as differential diagnosis generation and comprehensiveness of responses. Mixed methods are needed for more holistic evaluation.

Finally, the study uses LLMs that are trained using ML techniques. These models learn and adapt based on new data, which means their responses can change over time. While this allows the models to improve and become more accurate, it also introduces a level of unpredictability in their responses (Tredinnick & Laybats, 2023).

While this study generates useful insights, continued research with larger‐scale, multi‐site evaluations of both objective and subjective decision‐making measures is still warranted. The above limitations emphasize the need for additional validation of findings before generalizing applications of GenAI to complex medical contexts.

## 5 CONCLUSION AND IMPLICATIONS

This study offers valuable insights into the comparative performance of GenAI systems and experienced ICU nurses in terms of diagnostic accuracy and CDM. While some models matched or even surpassed human performance in standardized test cases, nurses consistently outperformed in complex, open‐ended scenarios that better represent real‐world clinical practice.

Our findings corroborate previous research, showing that while GenAI tools excel in narrowly defined tasks, they struggle in broader domains that require holistic clinical judgement. This highlights the ongoing challenge of generalizability for GenAI, a gap that may be bridged by experience‐based human evaluation. However, GenAI systems also demonstrated strengths, such as rapid response times, which could alleviate workforce constraints if judiciously integrated.

We observed performance variations across different GenAI systems and clinical scenarios. For example, a model that excelled in one case underperformed in others, underscoring the need for ongoing development of specialized medical knowledge and judgement capabilities in GenAI. We also noted differences in response formats, with GenAI explanations tending towards verbosity compared to the more succinct yet comprehensive responses of human practitioners.

These findings, while preliminary, provide a framework for rigorous analysis and continuous refinement of human‐GenAI synergies. Experience‐informed systems show promise in supporting less experienced clinicians and enhancing novice understanding through simulation, provided they are closely supervised. However, GenAI is not yet ready to autonomously replace core human clinical expertise.

Looking ahead, continued mixed‐method investigations involving diverse stakeholders can help iteratively develop safe and beneficial integrated care models. With an approach centered on augmenting rather than replacing human judgement, future GenAI systems may play a crucial role in partnership with clinicians to optimize complex medical decision‐making and improve patient outcomes.

Our study has several implications for theory, policy and practice in nursing. It underscores the potential of GenAI in enhancing CDM in nursing and points to the need for theoretical models that incorporate GenAI tools into nursing practice. These findings should inform policymakers in developing regulations around GenAI use in healthcare. One challenge identified in our study is the training of novice nurses. The integration of GenAI tools into their training could boost their decision‐making skills and confidence. However, it is vital to ensure that reliance on GenAI does not compromise the development of critical thinking and clinical judgement.

GenAI presents both opportunities and challenges in nursing. Opportunities include improved efficiency and accuracy in CDM, while challenges encompass ethical considerations, data privacy and the potential for over‐reliance on technology. These issues warrant further exploration in future research.

Rather than comparing the performance of ICU nurses and GenAI systems, we advocate for a complementary approach where GenAI supports CDM. GenAI can offer valuable insights and data, but the final decision should always rest with healthcare professionals who can take into account the patient's unique circumstances.

In conclusion, our study paves the way for further exploration of how GenAI can be integrated into nursing practice. Despite existing challenges, the potential benefits for patient care and nursing education are substantial. Future research should continue to explore these possibilities and address identified challenges. This integrated approach to GenAI and nursing has the potential to revolutionize patient care and transform nursing education.

## 6 RELEVANCE TO CLINICAL PRACTICE

This study offers preliminary but important perspectives on leveraging GenAI to support rather than replace the experience and holistic perspective that expert nurses bring to complex patient assessments in ICUs. With further development and evaluation, GenAI‐assisted decision support informed by this research could help reduce cognitive workload for nurses, facilitate second opinions and promote more explainable, consistent CDM—ultimately improving ICU patient outcomes. Continued dialogue between technology developers, clinicians and researchers will be essential to optimizing human‐GenAI synergy for critical care.

## FUNDING INFORMATION

The authors declare that no funds, grants, or other support were received during the preparation of this manuscript.

## CONFLICT OF INTEREST STATEMENT

The authors declare that they have no known competing financial interests or personal relationships that could have appeared to influence the work reported in this paper.

## ETHICS STATEMENT

Prior to commencement, the study obtained approval from the university's ethics committee (#0006223‐2). All data collection procedures ensured anonymity. Nurses offered informed consent before participating, with the assurance of their right to withdraw from the study at any point and for any reason.

## INFORMED CONSENT

There was no need to obtain informed consent from legal guardians.

## Supporting information


Data S1.

Appendix S1.

## Data Availability

The data that support the findings of this study are available from the corresponding author, upon reasonable request.
