# Supplementary material for: Augmenting intensive care unit nursing practice with generative AI: A formative study of diagnostic synergies using simulation‐based clinical cases
Source: J Clin Nurs. 2024 Aug 5;34(7):2898–907. doi: 10.1111/jocn.17384 (PMC12181158; doi:10.1111/jocn.17384)
Supplement: Supplementary file 2 — Appendix S1. [file JOCN-34-2898-s002.docx]

Appendix

Case 1: Cardiopulmonary Resuscitation.

A 51-year-old male patient, post anterior myocardial infarction, is admitted to the cardiology department and is fully monitored. While distributing medications, the nurse notices eye rolling and loss of consciousness. On the monitor, ventricular tachycardia is observed.

Q1. What is the immediate action the nurse should take?

Q2. What actions should be taken on the patient?

Q3. If there is no pulse, what should the nurse do next?

Two additional team members join with the resuscitation cart and a defibrillator. On the monitor, ventricular tachycardia is observed. While checking for a central pulse, the patient is connected to the defibrillator.

Q4. How should the nurse proceed?

After 5 more cycles, a central pulse is detected, and on the monitor, a sinus rhythm with a rate of 69 beats per minute is observed. The decision is made to continue treatment according to the ROSC (Return of Spontaneous Circulation) protocol.

Q5. What does this treatment include?
